# Role of supercritical carbon dioxide (scCO_2_) in fabrication of inorganic-based materials: a green and unique route

**DOI:** 10.1080/14686996.2021.1955603

**Published:** 2021-09-06

**Authors:** Hao Liu, Biao-Qi Chen, Yu-Jing Pan, Chao-Ping Fu, Ranjith Kumar Kankala, Shi-Bin Wang, Ai-Zheng Chen

**Affiliations:** a Institute of Biomaterials and Tissue Engineering, Huaqiao University, Xiamen, P. R. China; b College of Chemical Engineering, Huaqiao University, Xiamen, P. R. China; c Fujian Provincial Key Laboratory of Biochemical Technology, Huaqiao University, Xiamen, P. R. China

**Keywords:** Supercritical fluids, inorganic materials, aerogels, exfoliation, scCO_2_-assisted deposition, 70 New topics/Others, 106 Metallic materials

## Abstract

In recent times, the supercritical carbon dioxide (scCO_2_) process has attracted increasing attention in fabricating diverse materials due to the attractive features of environmentally benign nature and economically promising character. Owing to these unique characteristics and high-penetrability, as well as diffusivity conditions of scCO_2_, this high-pressure technology, with mild operation conditions, cost-effective, and non-toxic, among others, is often applied to fabricate various organic and inorganic-based materials, resulting in the unique crystal architectures (amorphous, crystalline, and heterojunction), tunable architectures (nanoparticles, nanosheets, and aerogels) for diverse applications. In this review, we give an emphasis on the fabrication of various inorganic-based materials, highlighting the recent research on the driving factors for improving the quality of fabrication in scCO_2_, procedures for production and dispersion in scCO_2_, as well as common indicators utilized to assess quality and processing ability of materials. Next, we highlight the effects of specific properties of scCO_2_ towards synthesizing the highly functional inorganic-based nanomaterials. Finally, we summarize this compilation with interesting perspectives, aiming to arouse a more comprehensive utilization of scCO_2_ to broaden the horizon in exploring the green/eco-friendly processing of such versatile inorganic-based materials. Together, we firmly believe that this compilation endeavors to disclose the latent capability and universal prevalence of scCO_2_ in the synthesis and processing of inorganic-based materials.

## Graphical abstract

**Figure:**
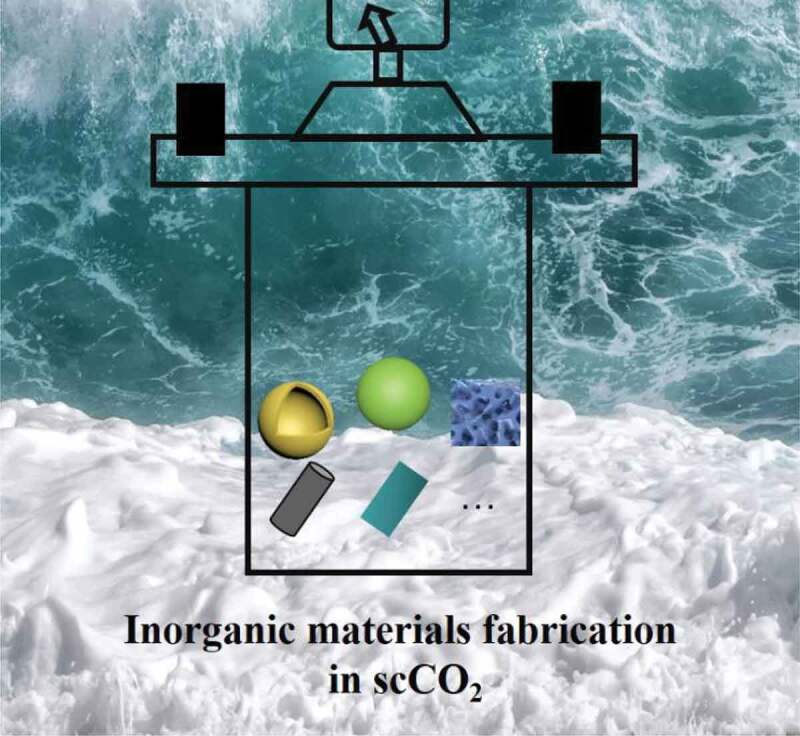

## 1. Introduction

Owing to their tunable morphological attributes and desirable physicochemical properties, various types of inorganic materials have garnered enormous attention from researchers and being more preferred compared to organic-based materials **[**1**]**. In addition, these inorganic-based materials can be conveniently fabricated in extensive subtypes by the combination of various metals of interest towards their utilization in diverse applications. In this vein, several classic examples include silica-based **[**2–4**]**, different transition metals-, and semiconductor-based constructs **[**5,6**]**, metal-organic frameworks (MOFs) **[**7**]**, as well as carbon nanotube (CNT)-based composites, among others **[**8**]**. Along this line, the innovative nanoarchitectures with distinctive morphologies, *i.e*. numerous dimensions of 0-dimensional materials (nanodots), 1-dimensional materials (nanorods, and nanowires), 2-dimensional materials (nanosheets), and 3-dimensional materials (spheres and hollow constructs) **[**9**]**, and porosity (porous as well as non-porous) offer higher performance efficiency compared to their bulk substrates, which are of particular interest in catalysis **[**10**]**, optics **[**11**]**, energy **[**12**]**, and biomedicine **[**13–15**]**. Accordingly, diverse methodologies have been employed to fabricate such innovative inorganic-based architectures, such as chemical precipitation **[**16**]**, sol-gel **[**17**]**, and hydrothermal **[**18**]**. However, it should be noted that the intrinsic physicochemical properties, and eventual performance of the designed resultant nanoarchitectures, are closely bound to their morphology in terms of particle size and shape of the eventual structures. The eventual morphological attributes depend on the regulation of the experimental conditions (reaction temperature, time, and stirring speed) and the alteration in the organic solvents (usually harmful) used during the preparation **[**19,20**]**. Therefore, several efforts have been dedicated to employing different methods and their hybrid combination towards producing such inorganic-based architectures through an environmental-friendly, rapid, and facile synthetic route.

To this end, the supercritical fluid (SCF) technology has emerged as a promising alternative in processing diverse materials of interest due to its exceptional processing convenience and environmentally benign character, as well as the economically advantageous nature of SCFs **[**21**]**. Moreover, several distinctive properties of SCFs include better diffusivity of SCFs over the organic solvents in fabricating the nanostructures and the continuously tunable, experimental conditions, as well as solution concentration. In some instances, the utilization of organic solvents is inevitable, which, however, can be removed eventually through flushing SCFs. In addition, SCFs offer gas-like and liquid-like characters, facilitating the convenience of handling any type of material efficiently while fabricating various carrier systems. More importantly, the non-toxic and non-inflammable features of SCFs have pushed the researchers towards their enormous applicability for establishing the eco-friendly preparation strategy. Owing to these exceptional characteristics, SCFs have been applied towards the fabrication of diverse organic- and inorganic-based architectures. Among diverse SCFs, supercritical CO_2_ (scCO_2_) has garnered enormous attention in fabricating various materials for diverse applications **[**22,23**]**. In this regard, scCO_2_ has been oftentimes utilized to process organic-based materials, for instance, polymers, while supercritical water (scH_2_O) has been applied to subject the processing of enormous varieties of inorganic-based materials towards fabricating fluorophores and other luminescent materials **[**24**]**. Although scCO_2_ has been extensively utilized in handling organic-based materials, in recent times, it has been extensively applied in the processing the inorganic-based materials for a wide range of applications, including but not limited to the material synthesis **[**25**]**, encapsulation of the desired metal-based nanoparticles into polymers and inorganic substrates (core-shell nanoparticles) **[**26**]**, selective extraction **[**27**]**, intercalation, and exfoliation of layered materials **[**28–30**]**, cleaning, as well as drying of meso- and microporous materials **[**31**]**, among others **[**32–34**]**.

Notably, the SCF-based process can act as both a bottom-up and a top-down type of particle fabrication approach. Accordingly, the changes in the critical conditions of the SCF processing, *i.e*. pressure and temperature, can affect the fabrication of innovative inorganic-based architectures through a reversible phase transition from crystalline to amorphous forms, as well as exfoliation of materials. However, it should be noted that, based on the optimum critical pressure conditions provided, the experimental operational dangers would be apparently reduced. On the one end, as a bottom-up approach, several types of nanocomposites produced by the scCO_2_-assisted processes from the atomic level to defined structures often result in improved performance compared to their substrates due to their highly advantageous morphological attributes and enriched physicochemical properties **[**25,35**]**. On the other hand, as a prototype of a top-down approach, the utilization of scCO_2_ can facilitate the exfoliation of the prepared materials, resulting in two-dimensional (2D) nanosheets. In the vein of fabricating diverse materials of interest, various scCO_2_-based processes can be applied to synthesize and activate inorganic-based structures; 1) the rapid expansion of scCO_2_ solution (RESS), scCO_2_ anti-solvent process (SAS) possess enormous abilities towards the fabrication of diverse inorganic-based nanomaterials with excellent catalytic performance; 2) scCO_2_-deposition process (SCD) for supporting structures; 3) scCO_2_-facilitated exfoliation of 2D materials, which usually bound to phase engineering and heterojunction fabrication; 4) the scCO_2_-based drying process (SCFD) to prepare the aerogels; and 5) the scCO_2_-based process to activate or fabricate metal-organic frameworks (MOFs). The resulting inorganic-based nanoarchitectures from diverse approaches often utilize the high dispersity of scCO_2_ towards special applications, including the preparation of large-volume capacitors and high-performance lubricants as well as biomedical products **[**36–39**]**.

**Figure 1.**
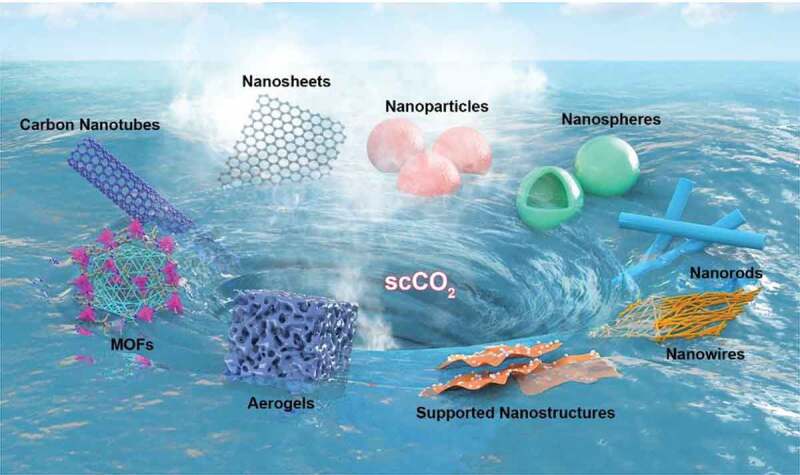
Schematic illustration showing the fabrication of diverse inorganic-based nanomaterials using the scCO_2_ technology

Despite the fact that several reviews have been published regarding the synthesis of diverse materials, most of the compilations from us and various groups globally have been focused on the fabrication of materials using different types of precursors, specifically organic molecules and polymers, in an arbitrary size range of macro-sized constructs to micro-sized particles, and sub-micron architectures as well as hybrid composites for different applications **[**40–42**]**. In this review, we comprehensively emphasize recent advances in fabricating various inorganic-based nanomaterials *via* scCO_2_ technology with a particular focus on the recently reported intriguing studies (Figure 1). Initially, we introduce the fabrication of the diverse inorganic species based on the several typical processes based on the scCO_2_ technology, highlighting the effects of various parameters in the fabrication towards diverse applications. Finally, we summarize the viewpoints and potential outlook of these innovative inorganic nanostructures.

## 2. Preparation of diverse inorganic-based materials

Owing to the distinctive and highly advantageous properties of scCO_2_, such as excellent diffusivity, incessantly tunable experimental (processing and formulation) conditions, the high-pressure-based technology can be conveniently applied towards the fabrication of diverse inorganic-based materials in terms of bottom-up and top-down ways. In this vein, several types of inorganic materials have been fabricated, including but not limited to nanoparticles-based on various inorganic metals with high dispersion, exfoliation of 2D layered materials by CO_2_ molecules, hollow nanospheres with high surface area and tunable structures, as well as materials with high porosity, such as aerogels and MOFs. In this section, we provide an emphasis based on using various prototypes of the scCO_2_-based technology in the fabrication of the aforementioned inorganic-based materials with a particular focus on the recently reported literature. In addition, several types of composites based on inorganic-inorganic (decorating nanoparticles-supported structure) and organic-inorganic (MOFs and polymeric composites with inorganic architectures) nanocomposites generated using these scCO_2_-based approaches are also discussed. Notably, the resultant products and their characteristics often result in improved performance, which, however, dependent on the aforementioned operating conditions.

### 2.1 Fabrication of inorganic-based nanoparticles/nanospheres

**Figure 2.**
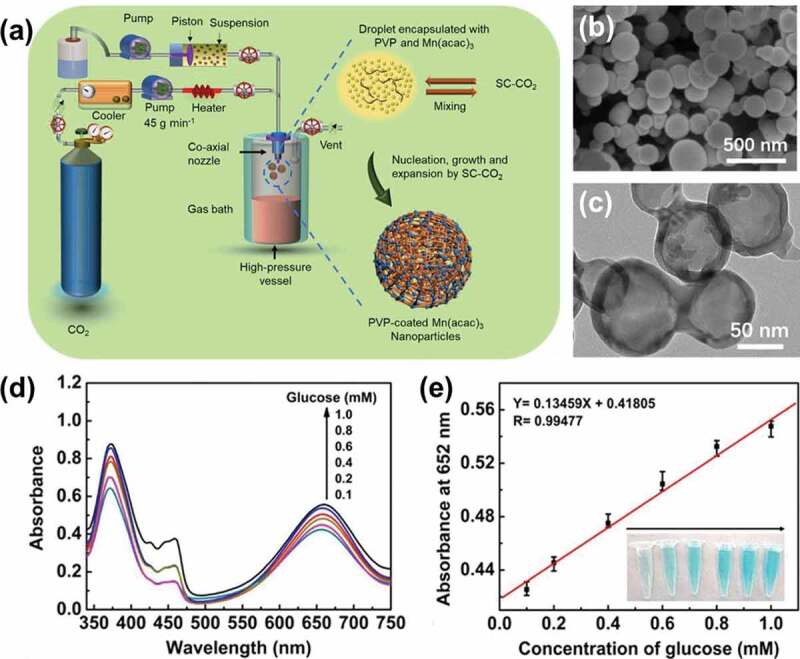
(a) Schematic of the instrument setup of the SAS technology and relevant mechanism of PVP encapsulated Mn(acac)3 nanoreactor formation. (b) FE-SEM image of as-prepared PVP-coated Mn(acac)3 nanoparticles; (c) TEM images of MHNs; (d) Linear standard curve for glucose determination. (e) the corresponding photograph of the solutions containing different concentrations of glucose. Reproduced with permission from Ref. [54], Copyright 2019, American Chemical Society

Despite the availability of various prototypes of the scCO_2_ technology, the conventional RESS and SAS approaches are indeed versatile and widely applied prototypes in the fabrication of the inorganic-based architectures, similar to the polymeric composites. Among these two predominant approaches, the generalized SAS process utilizes some of the organic solvents, such as methanol for acetylacetonate metal salts or acetone for nitrates, to dissolve the metal salt directly or metal-polymers mixture. Meanwhile, the liquid mixture is formed rapidly due to the low viscosity of scCO_2_ and extensive mass transfer rates between the SCF and the solution droplets at their point of contact. Further, the mixture is pumped through a heated nozzle into a low-pressure chamber (RESS) or sprayed through the coaxial nozzle (SAS), leading to the rapid nucleation or solution supersaturation and subsequent precipitation of the solute, resulting in the metallic nanostructures. Notably, solvent-induced phase separation acts as the guiding principle of this operating system. Moreover, the morphology of the resultant end-products, such as the particle size, shape of the nanoparticles, stringently depends on the experimental parameters such as temperature, pressure, and solubility of the added components in the solvent system. It should be noted that the final morphology of the resultant nanoparticles is closely related to their overall performance. In the SAS process, experimental factors like temperature, pressure, and the solubility of the solute in SCF, and the miscibility of SCF and organic solvent are needed to be optimized. The design of a liquid/solution injection device is another critical factor, which plays a vital role in the eventual output of the inorganic architectures. These consequences of optimized operations are responsible for the generation of a liquid jet break-up, resulting in the small-sized droplets due to the enhanced mass transfer between the liquid and the gaseous phases (Figure 2a). After the nanoparticle precipitation procedure, the washing process using the solvent mixture with scCO_2_ is often essential to stabilize the supercritical state, avoiding the liquid phase condensation, which not only influences the properties of inorganic nanoparticles and but also facilitates in overcoming the accumulation of the precipitate blocking the pipeline of the SAS device.

As mentioned earlier, several morphological and textural properties of the fabricated inorganic materials, in terms of smaller particle size and higher specific surface area, further promote the photocatalytic properties [43], catalysis ability [38,39], and even the biological performance of the nanostructures substantially [44]. Accordingly, several efforts have been dedicated to preparing inorganic-based nanostructures using the conventional RESS and SAS methods for enriched performance (Table 1). In this case, Sun and colleagues applied the RESS process by utilizing the water and scCO_2_ (w/c)-based microemulsion as a solvent system to improve the dissolution of silver nitrate (AgNO_3_) [45]. This highly soluble system was rapidly expanded in the sodium borohydride solution at 300 K, resulting in the uniform-sized Ag nanoparticles (AgNPs) with an average diameter of 7.8 nm. Further, an advancement of RESS was made by placing the reductive solution at the receiving end of the rapid expansion by adjusting the solution to be highly basic [46]. Compared to RESS, several efforts have been dedicated to applying the SAS approach extensively towards the fabrication of various inorganic-based nanoarchitectures. In a case, Miedziak and coworkers fabricated the nanocrystalline cerium oxide (CeO_2_) supported gold-palladium alloy catalysts utilizing the widely applied SAS method [47]. Compared to the products obtained from the conventional preparation techniques, this unconventional SCF-assisted SAS process resulted in the lesser polluting components with higher active oxidation catalytic performance. Tang and colleagues prepared nanocrystalline copper/manganese oxide (Cu/MnO_x_) catalysts for CO oxidation [39,48]. The authors demonstrated that the material was more significant than 2-fold as active per unit surface area compared to that of the traditionally prepared hopcalite catalysts. In addition, the harmful solvent residue levels were lesser than the other nanomaterials prepared by the conventional methods. In addition, Nesterov and coworkers proposed an approach for the synthesis of multicomponent Ni-Cu catalysts with silica-based frameworks using the SAS process. Moreover, this approach also addressed the agglomeration issue by various conventional preparation methods, resulting in the size reduction of the large-sized nanoparticles [49,50]. In an attempt to regulate the precipitation of reactants by the dense scCO_2_-based anti-solvent route, Hutching and colleagues had studied the fabrication of various types of inorganic nanoparticles, including titanium dioxide (TiO_2_) [51], CeO_2_ [52], copper/zinc oxide/aluminum trioxide (Cu/ZnO/Al_2_O_3_) as catalysts [53]. In this approach, the PVP-based inorganic nanoreactors were initially prepared *via* the PVP-metal blend to subsequently fabricating the corresponding metal constructs.

**Table 1.** Summary of the fabricated nanoparticles using conventional RESS & SAS processes

Metal/metal oxide nanoparticle	Precursor	Synthetic method	Particle size (nm)	Conversion method/agent	Ref.
Ag	AgNO_3_ and w/c microemulsion	RESS	7.8	Chemical reduction	[45]
	AgNO_3_ and w/c microemulsion	RESS	3.9–12	Chemical reduction	[46]
TiO_2_	TIP	SAS	316.8 ± 2.7	Hydrolysis reaction	[51]
CuMn_2_O_4_	Mn, Cu nitrate and Na_2_CO_3_ aqueous solution	SAS	160–200	Calcined in air (400°C, 2 h)	[39,48]
Co_3_O_4_/ZnO	Zn, Co acetate and H_2_O/methanol solution	SAS	-	Calcined in air	[125]
Cu/ZnO/ Al_2_O_3_	Cu, Zn, Al acetate and ethanol-water solution	SAS	-	Calcined in air (300°C, 4 h)	[53]
Ce_1 −x_Zr_x_O_2_	Ce(acac)_3_, Zr(acac)_4_ and methanol solution	SAS	30–50	Calcined in air (600°C, 2 h)	[57]
CuO-CeO_2_-ZrO_2_	Cu acetate, Ce(acac)_3_, Zr(acac)_4,_ and methanol solution	SAS	9.9–12.8	Calcined in air (600°C, 2 h)	[54]
Ni–Cu	Ni, Cu acetates and methanol solution	SAS	< 10	Calcined in air (300°C)	[49,50]
MnO_x_–CeO_2_	PVP, Mn, and Ce(acac)_3_ methanol solution	SAS	-	Calcined in air (600°C, 2 h)	[55,56]
AlOOH@ RGO hybrids	Al(NO_3_)_3_^.^9H_2_O and ethanol solution	SAS	Nanosheets thickness:12.6	High temperature treatment:140°C	[126]
MHNs	PVP, Mn(acac)_3_ and methanol solution	SAS	250	Calcined in air (600°C, 2 h)	[36]
ZnO, NiO, and Co_3_O_4_	PVM/MA, metal nitrates and acetone solution	SAS	25.8 ± 8.0, 23.3 ± 7.9, and 28.5 ± 7.5	Calcined in air (600°C, 2 h)	[64]

***Abbreviations****: Ag: silver; Al_2_O_3_: aluminum trioxide; AlOOH: aluminum oxide hydrate; CeO_2_: cerium dioxide; Co_3_O_4_: cobalt tetraoxide; CuMn_2_O_4_: copper-manganese oxide; CuO: copper oxide; MHNs: Mn_2_O_3_ hollow nanoparticles; NiO: nickel oxide; PVM/MA: poly- (methyl vinyl ether-co-maleic anhydride); PVP: poly(N-vinyl-2-pyrrolidone); RGO: reduced graphene oxide; TiO_2_: titanium dioxide; TIP: Titanium (IV) isopropoxide solution; w/o microemulsion: water and scCO_2_ microemulsion; ZnO: zinc oxide; ZrO_2_: zirconium oxide.*

Owing to their unique structural arrangement and advantageous properties, hollow nanoarchitectures have garnered enormous attention from researchers in various fields of catalysis, biomedicine, biosensors, and chemical sensors, among others [10,36,54]. In regards to the remarkable surface characteristics and formation mechanism of nanoparticles, hollow nanomaterials are proved to be one of the most renowned nanoarchitectures, especially in terms of developing synthesis routes and structure-related physicochemical properties [51,52,54]. There exist several approaches in fabricating these inorganic-based hollow matrices, such as self-assembly, templating using silica and micelles, as well as chemical etching methods, among others. Most of these conventional methods suffer from several shortcomings of numerous operation steps and the utilization of harmful organic solvent residues in the end product. In this vein, several efforts have been dedicated to addressing these problems while fabricating hollow nanoarchitectures. In an instance, taking advantage of the SAS method, Jiang and colleagues successfully prepared the diverse hollow bimetallic and polymetallic oxides-based nanospheres, based on MnO_x_-CeO_2_ [55,56], Ce_1−x_Zr_x_O_2_ [57], and CuO-CeO_2_-ZrO_2_ [54]. These studies demonstrated that the addition of a surface-active agent could lower the surface tension of the droplets, improving the dispersion of fabricated metal-oxide nanoparticles. In addition to PVP, various other surfactants, such as cetyltrimethylammonium bromide (CTAB), Tergitol (TM)XH(Nonionic) (P123), Poloxamer (F127), and poly(ethylene glycol) (PEG), have been utilized. These templates could enhance the dispersity of the MnO_x_-CeO_2_ bimetallic nanoparticles, guiding their rearrangement during the fabrication. The experimental results proved that P123 and PVP evidently decreased the undesired interactions and aggregation attributes of the metal oxide nanoparticles. Moreover, the resultant inorganic nanoparticles with high dispersion ability possessed a larger specific area compared to their counterparts. Notably, the most representative type of nanoconstructs among all the resultant inorganic nanoparticles is the spherical-shaped nanoconstructs (Figure 2). These consequences indicated that the resultant nanostructures produced by the SAS approach were more distinctive and effective compared to other prototypes of the scCO_2_-based fabrication technology. All these exceptional features led to higher catalytic properties of MC-P123 and MC-PVP for the low-temperature deNO_x_ in the existence of NH_3_. Recently, Chen’s group applied a similar approach to fabricating MnO_2_ nanospheres by the SCF-assisted PVP nanoreactors to detect glucose effectively (Figure 2a) [36]. The MnO_2_ nanozymes showed the dual-enzyme property in the presence of TMB as a substrate, resulting in efficient catalytic performance (Figure 2b). The colorimetric results exhibited an excellent performance toward determining glucose levels within a wide linear range and a limit of detection (LOD) of 2.31 μM (Figure 2(d,Figure 2e)). Compared to the other reported materials for existing other peroxidase-mimicking materials, these MHNs by the SAS approach revealed enriched glucose catalytic activity.

Recently, inorganic nanoparticles with favorable conductivities and light absorption have attracted significant attention from researchers towards improving the performance of various first-row transition metals. Nevertheless, most inorganic nanoparticles exhibit no strong absorption of visible light because of their large bandgap and small optical intersecting surface. To overcome this limitation, an effective approach doping the non-metallic elements into the inorganic lattice can be applied, which in turn further improves the light absorptivity of the nanoparticles [58]. The doping behavior happens to be favorable by forming intermediate energy levels and broadening of the valence band, causing intensified visible light absorption. In this vein, numerous advancements have been reported in the improvement of visible light absorption and broader visible light absorption range *via* integration of carbon [59], sulfur [60], oxygen [61], and nitrogen [62,63], in the inorganic nanostructures. For the metal oxide-based architectures, doping the non-metallic elements into the metal oxide lattice is an active strategy to raise the light absorptivity of the nanoparticles, as the elements are a comparatively larger size than lattice oxygen but with lower electronegativity [56]. The doping behavior brings about the formation of intermediate energy levels and extension of the valence band, leading to enhanced visible light absorption. Incorporation of the non-metallic elements in many wide-band-gap metal oxides improves their intrinsic conductivities and creates a channel for separation of photo-generated electrons and hole. Owing to the ease of solubility of PVM/MA in acetone and its stable properties and non-reactivity with metal nitrates, Jiang and colleagues fabricated the multiple C-doped metal oxide nanoparticles *via* combining the SAS method with the thermal annealing of metal nitrates [64]. Initially, metal oxide precursors, such as Co(NO_3_)_2_, Ni(NO_3_)_2_, and Zn(NO_3_)_2_ were loaded in PVM/MA, resulting in the polymeric nanoreactors. Further, the morphology of the resultant processed nanoreactors could be conveniently altered by regulating the processing and formulation parameters. Notably, the coating of PVM/MA over the metal nitrates provided an opportunity for well-proportioned incorporation of carbon in the metal oxides, avoiding non-homogeneous doping of carbon species and aggregation of the metal oxide nanoparticles. Further, the experimental results indicated that an increase in the concentration of carbon doping resulted in the enhanced absorption of C-doped NiO and ZnO nanoparticles in the visible light region over the low concentration of carbon doping. In addition, the metal oxide nanoparticles with the C doping possessed the photoinduced antibacterial capability. Amongst all the composites, the ZnO nanoparticles with a high carbon doping level offered improved antibacterial ability. To this end, the photothermal conversion efficiency of carbon-doped Co_3_O_4_ nanoconstructs was highly commendable. Therefore, this innovative SAS method could be available in the production of high-performance structures and materials built on inorganic nanoparticles. However, the inorganic nanoparticles prepared by the SAS method often suffer from a major disadvantage of poor dispersion ability, significantly limiting its applicability in various fields. In addition, the choice of solvent and the influence of processing parameters substantially play significant roles in the particle properties. Therefore, the effect of scCO_2_ on surface shape and volume shape factors, as well as particle dispersion ability should be the focus in the future research.

### 2.2 Decoration of inorganic nanoparticles

In addition to the fabrication of various inorganic-based constructs, it is convenient the generate various inorganic component-supported nanoparticles [65] and films [42] using the SCF-assisted deposition (SCD) process. Briefly, the inorganic precursors are initially dissolved in scCO_2_, and the nanoparticles are then adsorbed onto the intended substrates. Nonetheless, the successful transformation of the inorganic precursors to the corresponding metal or metal oxide nanoconstructs is often achieved by using *in situ* reduction or oxidation processes. In this regard, it is viable to disperse inorganic nanoparticles or precipitate coating films on many organic or inorganic materials employing this SCD method. Notably, the conversion procedure is perhaps caused due to various reasons, such as applying high-temperatures, adding some conversion reagents (hydrogen or ethanol solution in scCO_2_) [66], by setting the system at low pressure after the depressurization [67], and using the method of laser irradiation [68,69]. The conversion method can dictate the dispersion efficiency of the formed nanoparticles, films, or rods while loading on the substrates. However, multiple steps are required for the effective deposition of the inorganic precursors on the substrates using the SCF-assisted processes. In general, the dissolution of the metal precursor in scCO_2_ can be deemed to be the start of the SCD process. Some inorganic salts have been employed, such as metal-based chelates and organics. In this context, enormous efforts have been dedicated to exploring the advancements in terms of the solubility of inorganic complexes with ligand types in scCO_2_. Employing semi-empirical correlations or equations of state-based methods can yet be regarded as an appropriate way to predict the solubility of inorganic complexes in scCO_2_ towards achieving substantial deposition efficiency [70,71].

**Figure 3.**
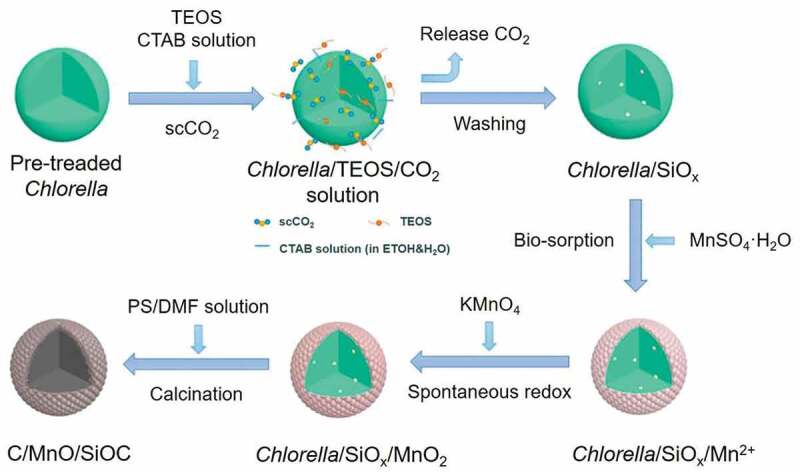
Schematic illustration of the synthesis process of C/MnO/SiOC composites. Reproduced with permission from Ref [80]. Copyright 2019, Elsevier

The second step of the SCD process is the inorganic precursor adsorption on the substrates. For some substrates having a large specific surface area, like carbon nanotubes, graphene oxide sheets, graphene, the inorganic complexes can be deposited on the surface of solid-state structures. In this context, the adsorption thermodynamics of the inorganic precursor onto the particular substrate plays a crucial role in the SCD-assisted process, which has a direct correlation relationship with the concentration of the inorganic precursor in the scCO_2_ phase on the adsorption isotherm. It might be one of the reasons why the amount of the inorganic precursor absorbing on the particular substrate could confirm through the adsorption isotherms. Hitherto, some studies were reported concerning the fundamentals of adsorption thermodynamics of the precursors loaded onto the supports using scCO_2_. These studies included the facile adsorption of some precious metal complexes on the surfaces of the carbon-related multi-aperture materials [72–74]. Comparatively, the SCD process is certainly different, in which the altered parameters and their preferences can affect the solute dissolving capacity. As the liquid inorganic precursors are inclined to transform to solid at conditions of low pressure, in temperature reliability, and particular precursor concentration, the adsorption rate of the inorganic nanoparticle onto the substrates is absolutely improved. Concerning the influence of the temperature on the absorption rate of inorganic precursors onto the substrates [75,76], the positive correlation of temperature and increase in the adsorption of the precursors is observed. However, in some cases, it is deemed to be competitive adsorption between the solute and CO_2_ on the surface of the substrates [77–79]. In an attempt to improve the excellent deposition efficiency (Figure 3), a template, Chlorella, was applied in the typical SCD process not only as a source of carbon but also to facilitate as a biological template in fabricating the SiOC microspheres with simulated morphology. By taking advantage of the high diffusion rate and permeability attributes of scCO_2_, the substrates (CTAB and TEOS) could quickly liquefy and diffuse into the interlamination and gaps of Chlorella, attaining homogeneous distribution of TEOS in Chlorella. After TEOS hydrolysis, the mixture (Chlorella/CO_2_/TEOS) would be adapted into evenly distributed SiO_x_/Chlorella precursors. Then, the rice-like manganese oxide (MnO) nanoparticles were tightly embedded in the SiOC matrix by the Chlorella-based bio-sorption process, providing an effective strategy to prepare high capacity SiOC-based anode materials for efficient Li-ion batteries [80]. In this vein, several efforts have been dedicated to using the SCD method towards the precipitation of the nanoparticles or films of inorganic architectures like colloidal particles [74], carbon nanotubes [8], silica-based matrices [81], and aerogels or xerogels [82,83]. Various metallic substrates with exceptional properties include nickel (Ni), copper (Cu), platinum (Pt), silver (Ag), palladium (Pd), cobalt (Co), and gold (Au). Table 2 summarizes some recently reported studies based on the SCD process.

**Table 2.** Summary of the supported metal nanostructures synthesized using the scCO2 deposition process

Metal/metal oxide nanoparticles	Substrate	Solvent medium	Particle size (nm)	Conversion method/agent	Ref.
Pb	SBA-15	scCO_2_	13.7	H_2_	[65]
	γ-Alumina	scCO_2_	2.9 ± 1.7	H_2_	[66]
	CNC	scCO_2_	6–13	Cellulose nanocrystals	[127]
	Porous SiO_2_/TiO_2_	scCO_2_	~3	H_2_	[81]
	CNT	scCO_2_	3–5	H_2_	[128]
	MWCNT	scCO_2_	7–9	H_2_	[129]
Pt	CNT	scCO_2_	6.9 ± 2.6	H_2_	[85]
	GA	scCO_2_	1.2–2.9	Thermal reduction	[82]
	GNP, CB and their hybrids	scCO_2_	1.7–2.4	N_2_/Thermal reduction	[130]
Au	GO	scCO_2_ +_ _ethanol	4–10	Chemical reduction	[37]
Ag	SA	scCO_2_	2–4	Laser irradiation	[69]
	MWCNT	scCO_2_+ ethanol	10–100	Chemical reduction	[84]
Cu	CNT	scCO_2_ + methanol	2–5	H_2_	[86]
Ni	Kevlar fabric	scCO_2_	200–250	Electroless plating	[131]
CuO	SBA-15	scCO_2_ + ethanol/ water	2.9 ± 1.0–6.5 ± 2.9	Calcined in air	[132]
Fe_3_O_4_	HPCs	scCO_2_ + ethanol	12.1–17.6	Calcined in air	[133]
Co_3_O_4_	SBA-15	scCO_2_ + ethanol	-	Calcined in air	[127]

***Abbreviations****: Ag: sliver; Au: gold; CB: carbon black; CNC: cellulose nanocrystals; CNT: carbon nanotubes; Co_3_O_4_: Preparation of Superhighly Dispersed Co3O4@SBA-15 with Different Morphologies in Supercritical CO2 with the Assistance of Dilute Acids. DOI:10.1021/ie501241f. cobalt tetraoxide; Cu: copper; CuO: copper oxide; Fe_3_O_4_: iron (ii, iii) oxide; GA: graphene aerogel; GNP: graphene nanoplatelets; GO: graphene oxide; HPCs: hierarchical porous carbons; MWCNTs: multi-walled carbon nanotubes; Ni: nickel; Pd: palladium; Pt: platinum; SA: silica aerogel.*

**Figure 4.**
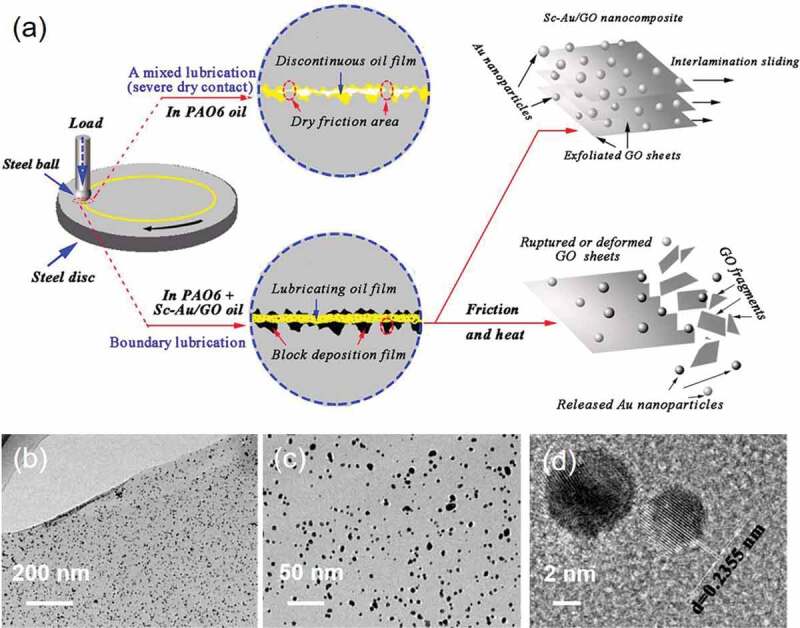
A) Schematic of the lubricating models of the pure PAO6 oil and the Sc−Au/GO dispersed oil for the ball-on-disc friction tester; Representative TEM images (b, c) Sc–Au/GO; and (d) a typical HR-TEM image of Au nanoparticles on GO sheets from Sc–Au/GO. Reproduced with permission from Ref. [81] Copyright 2019, American Chemical Society

In some instances, several zero-dimensional nanodots (AgNPs and AuNPs) have been deposited as supporting structures onto various substrates to improve the performance efficiency of the parent substrates, for instance, as lubricant additives and antimicrobial components. In contemporary industrial approaches, reducing the degree of wear has become a key factor in improving their durability and performance efficiency in most mechanical components. The utilization of lubricants has been widely recognized for improving the performance of machines. In addition to conventional organics, many metals- and metal oxide-based nanoparticles, graphene and graphite, as well as many other layer materials, have been shown to be operational lubricant additives for decreasing friction. The unique anisotropic crystal structure provides layered materials with strong covalent intra- and weak inter-layer interactions, ultimately leading to effective lubrication. Owing to the high diffusivity of scCO_2_, uniform deposition of nanoparticles onto any substrate can significantly improve the lubrication efficiency of the material. Accordingly, Su and coworkers applied the SCD technology to fabricate Au/graphene oxide nanocomposites and nano-Ag/MWCNTs nanocomposites, which could act as lubricants (Figure 4) [37,84]. Au/GO nanocomposites were added in the poly alpha olefins (PAO6) oil the friction factor, resulting in the reduction of wear rate up to 33.6 and 72.8%, respectively, towards exhibiting exceptional lubrication performance. Further, the friction factor and wear trace diameter were significantly reduced in engine oil diffused with 0.18 wt% of nano-Ag/MWCNTs nanostructures.

Finally, the reduction of the inorganic precursor to its metal elementary substance form (or oxidized to metal-oxide form) is the last procedure of the SCD process after solubility and deposition of the surface. The eventual properties of the supported nanoparticles and films in the end-product (for instance, particle size, distribution range, thickness of the film, and morphology) are often dependent on the reduced or oxidized forms of the nanoparticles and the processing conditions (reaction temperature and time). Typically, the first reduction of the metal precursor is performed using the reducing agents, such as the H_2_ reducing agent added to scCO_2_ and the metal precursor in contact with the substrate. In this vein, Rincón and colleagues fabricated diverse nanoparticles, such as Pb, Pt, and Cu-supported onto the carbon nanotubes by reducing the inorganic precursor using H_2_ [67,85,86]. Contrarily, in the case of the metal precursor being oxidized, the precursor organic solution was usually added dropwise to scCO_2_. Accordingly, previous reports indicated the utilization of different metal oxide nanoparticles supported onto various core substrates. In a case, Cheng and colleagues prepared the NiO nanoparticle-supported vertically-aligned carbon nanotube arrays (VACNTs) through the scCO_2_-assisted deposition after the annealing approach [87]. The nickelocene precursor was deposited in the gaps of VACNTs, demonstrating that the VACNTs/NiO composite arrays could result in a high capacitance of 1088.44 F·g^−1^. In another case, Rybaltovsky and coworkers explored the influence of laser irradiation at different wavelengths on the supported Ag nanoparticles generated using the SCD process and the Ag nanoparticles deposition behavior on silica aerogel [68,69]. The Ag precursor of the Ag(hfac)COD disposed of through the laser irradiation, demonstrating that the minimum size of the particles would be obtained using off-resonant irradiation, and the irradiation dosage could tune the particle concentration.

In virtue of regulating supported nanoparticle size and excellent dispersion property, the SCD process is currently one of the popular methods in fabricating inorganic nanocomposites. It should be noted the preferential crystalline features of various inorganics can be attained using arrested growth techniques. In this context, the inorganic precursor and stabilizers are dissolved in scCO_2_, and then the precursors are reduced to inorganic nanoparticles by employing several reducing substrates [42]. The stabilizers are then aimed to weaken the electrostatic neutralization or the steric hindrance of the nanoparticles. These consequences often yield better dispersion nanoparticles over the process without stabilizers in the scCO_2_ phase. For instance, a combination of methods (for instance, laser-assisted SCD synthesis) can also be employed in the fabrication of inorganic nanoparticles or films. However, it should be noted that the changes in the experimental conditions such as temperature, pressure, reagent concentrations substantially influence the properties of the nanoparticles, evidently consisting of a material medium, particle size and distribution, formation mechanism, and morphology. Considering these aspects carefully and strict optimization of parameters, this approach also results in new scCO_2_-assisted methods for the fabrication of diverse types of inorganic-based nanoarchitectures. Despite the success, the deposition rate using the SCD approach is not exceptionally high, similar to all chemical deposition methods. Moreover, the nanoparticles are often needed to be deposited locally. In addition, the deposition of the guest species on certain surfaces is always challenging, leading to poor performance efficiency of the nanocomposites. In some instances, the reactions involving in the guest species deposition yield residual gases after the reaction, which are often flammable and toxic, requiring stringent measures to prevent environmental pollution.

### 2.3 Exfoliation of 2D structures

**Figure 5.**
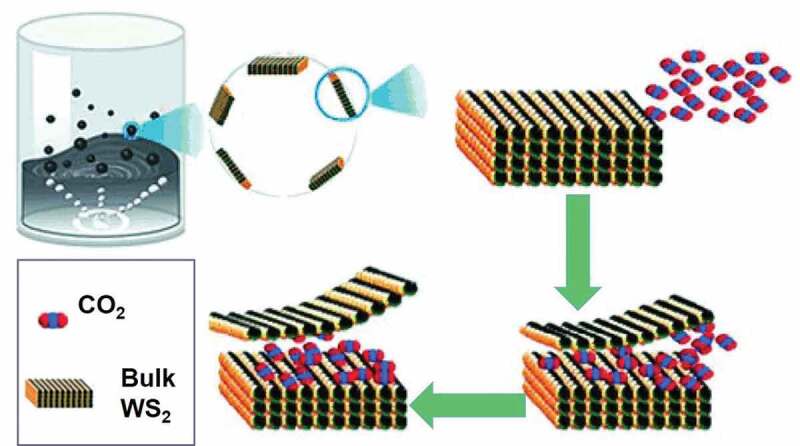
Schematic illustrating the scCO_2_ exfoliation of WS_2_ nanosheets. Reproduced with permission from Ref [92]. Copyright 2019, John Wiley & Sons

In virtue of the rise of highly productive techniques towards establishing the eco-friendly environment, scCO_2_ with such distinctive properties can be applied as an excellent medium for implanting and exfoliating layered 2D nanomaterials to prepare and process single- and few-layered nanosheets. Although the 2D multilayer structure is highly engaging and possesses the potential for diverse, innovative applications, monolayer or few-layer nanosheets produced under further exfoliation of the bulk structures offer excellent physicochemical properties and unique performance attributes, stringently relying on their atomic-layer thickness and 2D sheet-like morphology. In this vein, several advancements have been evidenced in exploring different preparation methods to fabricate the ultrathin 2D nanoplates. Among the various existing methods available, the liquid-phase exfoliation approach has been one of the often preferred top-down approaches for efficiently manufacturing thin nanosheets [88–91]. However, this top-down approach sometimes may result in the production of nanosheets with uneven thickness and large size distribution due to aggregation. To this end, scCO_2_ has been employed to assisting the exfoliation of layered materials, because of advantageous properties, like high diffusivity, distinguished surface wetting capacity, and low viscosity, as well as surface tension. In addition, the density and solvent strength of the SCFs can be adjusted from ‘gas-like’ to ‘liquid-like’ easily by regulating the critical conditions of pressure and temperature [25]. Moreover, it should be noted that the changes in the supercritical state of CO_2_ are closely related to the exfoliation process. For instance, the high diffusivity and vanishing surface tension of SCFs might enable the penetration of small fluid molecules into the interlayer spacing in WS_2_ bulks and assist in enhancing the exfoliation into high-quality single-layered WS_2_ nanosheets (Figure 5) [92]. In addition, the utilization of SCFs is often referred to as eco-friendly in nature as they are non-flammable and non-toxic, dissimilar to conventional exfoliation approaches applying high-toxic organic solutions, which may result in adverse environmental impacts. Considering these attributes, the scCO_2_-assisted liquid exfoliation has been investigated due to its eco-friendly processing and economical in nature, as well as safe handling of SCFs. In this sub-section, we emphasize the progress in the exfoliation of the 2D inorganic nanomaterials produced with the participation of scCO_2_, combining scCO_2_-based liquid-phase exfoliation, phase transformation, the establishment of amorphous inorganic materials, and novel functional nanomaterials, as well as the fabrication of heterostructures.

Interestingly, scCO_2_ offers controllable solvation strength and favorable surface tension, soaking, as well as unique transport characteristics, which made them an unparalleled and possibly excellent solvent medium for the effective exfoliation of 2D nanosheets. In an attempt to demonstrate the high exfoliation efficiency of the scCO_2_, Gai and colleagues employed a more-wall stator in water, scCO_2,_ and N-Methylpyrrolidone (NMP). The experimental results demonstrated that the yield of exfoliated nanosheets increased from 22% to 38% for NMP, from 18% to 33% for supercritical CO_2_, and from 17% to 27% for water with an increase in the shearing speed. The surface tension near the graphene and its higher viscosity caused an increase in the shear stress and reduction in the exfoliation energy, leading to the highest yields in the NMP solution. However, it should be noted that NMP is not an appropriate choice for exfoliation as it’s poisonous and highly challenging to remove the residues in the layers. Therefore, considering these aspects, the utilization of scCO_2_ offers more advantages over NMP and water regarding the fabrication of graphene and its application [28].

**Figure 6.**
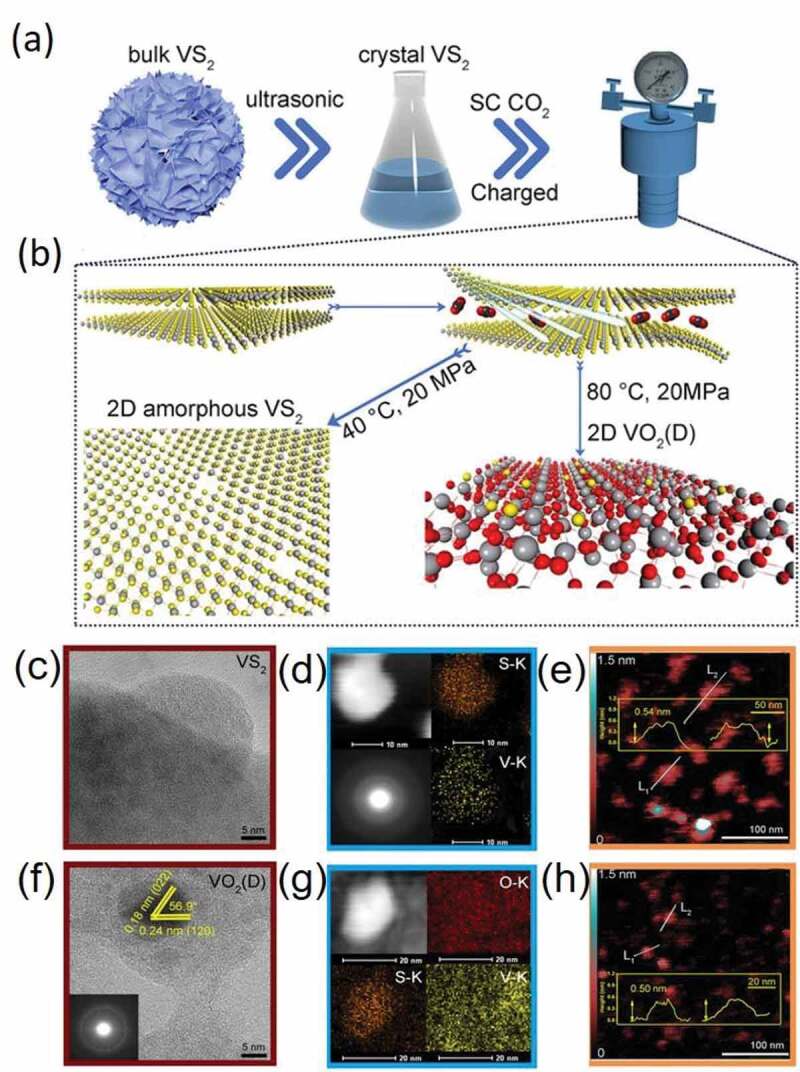
(a) Manufacturing process 2D S-doping VO2(D) with the assistance of scCO2 device. b) Molecular illustration of 2D amorphous VS2; c), f) HRTEM images of 2D amorphous VS2 and 2D VO2(D), (d), (g) STEM images and corresponding elemental mappings, (e), (h) AFM images of 2D amorphous VS2 and 2D VO2(D). Reproduced with permission from Ref. [99] Copyright 2019, John Wiley & Sons

In general, the 2D nanosheets fabricated using the scCO_2_-assisted exfoliation offer higher catalytic activity and reactive sites over other solvents. In a case, Zhou and colleagues demonstrated that the stimulation of the crystal alteration in 2D materials could create more active, reactive sites, promoting the initial activity of electrocatalysts for CO_2_ reduction [93]. These exceptional characteristics put forward an actual process employing scCO_2_ and water as a solvent mixture to producing the bismuth oxychloride (BiOCl) distorted interlamination architectures of 2D layers with diverse thicknesses. Theoretical simulation and experimental data indicated that the interlamination [Bi_2_O_2_]^2+^ crystal alteration would result from the diminished interlayer Cl atoms accomplished by the scCO_2_ phase transition. The energetics analysis confirmed that the distorted BiOCl nanosheets resulted in the extended intralayer structural distortion compared to that of the ultrathin nanosheets at the identical Cl defect. Consequently, it could effectively overturn the generation of CO and H_2_, as well as assure the conversion of CO_2_ to formate at exceptional selectivity (92%) and broad potential (−0.6 to −0.9 V). Moreover, they confirmed the easily tunable preparation process and mechanism of independent vanadium disulfide (VS_2_) nanosheets by simply varying the critical conditions (pressure and temperature) of scCO_2_ [94]. In this approach, monolayer VS_2_ nanosheets and 2D vanadium dioxide (VO_2_)(D) nanosheets with S‐doping *in situ* were attained at varied conditions in a controlled manner. As a result of 2D amorphous engineering transformation, the enhanced optical properties of VS_2_ nanosheets, 2D amorphous VS_2,_ and partially crystallized 2D VO_2_(D) were calculated as 42.4, 64.1, and 78.8%, respectively. Moreover, the highly crystalline quantum dots in VO_2_ (D) could improve heat transfer rate and photothermal conversion efficacy (Figure 6). Accordingly, with the help of scCO_2_, several advancements have been successfully made in reporting some complex heterostructures (WS_2_/WO_3_·H_2_O,2 H@1 T-MoS_2_/graphene, Ag/a-WO_3-x_) [95–97]. A summary of 2D materials exfoliated in scCO_2_ alone and some mixture solvent is provided in Table 3. Although the scCO_2_-assisted exfoliation shows promising potential, the relatively poor yield and the lack of clarity of the precise reaction mechanism limit its application. Although the product yield of scCO_2_-assisted exfoliation is higher than the conventional solution-based method, it is inadequate for practical applications. Therefore, the corresponding technology needs to be improved further. Apart from the basic physicochemical properties of scCO_2_, there is a little detailed explanation or mechanism for the typical effects, such as the enhanced strain effect on two-dimensional materials. For a better and deeper understanding of the mechanisms, a combination of extensive comparative experiments, the establishment of advanced characterization strategies, and theoretical calculations must be performed in subsequent studies.

**Table 3.** Summary of 2D materials exfoliated using the scCO_2_-assisted exfoliation process

2D materials	Exfoliation conditions	Solvent medium	Dimensions	Ref.
Graphene	Ultrasonication (300 W): 30 min; 8 MPa, 40°C in SCF; CF: 1500 rpm, 60 min.	ScCO_2_	Thickness: 0.44–0.61 nm Lateral size: 50–100 nm	[134]
	Ultrasonication (120 W) with 12 MPa, 40°C in SCF, 60 min	ScCO_2_	Thickness: 1–3 layers Lateral size: 0.5–5.0 µm	[34]
	15 MPa, 45°C in SCF, 30 min.	ScCO_2_	Thickness: 1.0–6.0 nm Lateral size: 0.2–1.0 µm	[135]
VS_2_	Ultrasonication: 2 h; 20 MPa, 40°C, 6 h in SCF; CF: 9000 rpm, 10 min.	Water/ scCO_2_/NMP	Thickness: 1 layer Lateral size: 1.7 ± 0.26 nm	[94]
BiOCl	20 MPa,160°C, 3 h in SCF, CF: 8000 rpm, 10 min.	Water/ scCO_2_	Thickness: 5 layers	[93]
WS_2_	Ultrasonication in an ice bath: 2 h; 16 MPa, 40°C, 3 h in SCF, CF: 3000 rpm, 15 min	Ethanol/water-scCO_2_	Thickness: 1 layer	[92]
MoS_2_	Ultrasonication: 6 h, 15 min; 80°C, 6 h in SCF.	ScCO_2_	Thickness: 1–3 layers Lateral size: 50 − 150 nm	[95]
	16 MPa, 75°C under shearing (1200 rpm), 3 h, in SCF; then ultrasonication, 30 min in ethanol, CF: 2000 rpm, 30 min.	ScCO_2_	Thickness: <10 layers (95%), 1–4 layers (50%)	[136]
MoO_3_	Ultrasonication in an ice bath: 3 h; 8–16 MPa, 40°C, 3 h in SCF; then sonicated for an additional 2 h, then 3000 rpm, 45 min.	Ethanol/water-scCO_2_	Thickness: Single or few layers Lateral size: 10–200 nm	[137]
	Ultrasonication: 60 min; 16 MPa, 80°C, 3 h in SCF, CF: 6000 rpm, 15 min.	Ethanol/water-scCO_2_	Thicknesses: 3–4 nm (2–3 layers) Lateral size: 140 nm	[138]
Amorphous MoO_3_ QDs	Ultrasonication: 2 h; 17 MPa, 40°C, 3 h in SCF, CF: 6000 rpm, 15 min.	Ethanol/water/30% H_2_O_2_-scCO_2_	Smallest lateral size: 4 nm	[139]
Co-MoO_3-x_	Ultrasonication in an ice bath:8 h, CF: 3000 rpm, 45 min; mixed with cobalt nitrate, 10 MPa, 100°C slowly elevated to 200°C, 6 h in SCF; CF: 5000 rpm, 10 min.	ScCO_2_	Lateral size: 3–5 nm	[140]
Ni_0.125_MoO_3_	Ultrasonication in an ice bath:8 h; CF: 3000 rpm, 45 min; mixed with nickel nitrate, 10 MPa, 100°C slowly elevated to 200°C, 6 h in SCF; CF: 5000 rpm, 10 min.	ScCO_2_	Lateral size:16.0 ± 1.0 nm	[141]
BN	10 MPa, 45°C in SCF, under ultrasonication (60 W), 40 min.	ScCO_2_	Thickness: <5 layers (90%), 1 layer (20%), 2 layers (40%) Lateral sizes: 0.5 − 2 µm	[30]

***Abbreviations****: BiOCl: bismuth oxychloride; BN: boron nitride; CF: centrifugation; scCO_2_: supercritical carbon dioxide; SCF: supercritical fluids; MoO_3_: Molybdenum Trioxide; MoS_2_: molybdenum disulfide; NMP: N-Methyl pyrrolidone; VS_2_: vanadium disulfide; WS_2_:* tungsten disulfide.

### 2.4 Inorganic-based aerogels

Indeed, scCO_2_ offers comprehensively distinctive properties, which can be explicitly used to synthesize and activate new types of materials, such as aerogels (metal or metal oxide aerogel, some polymer aerogels, aerogels particles) [47,69,76,98,99]. The process of sol-gel-based synthesis generally begins with the formation of a pseudo-solution through a hydrolysis and condensation reaction by blending some chemically active precursors. After gradual condensation, the colloidal dispersion results in the formation of a holonomic meshwork, in which the solvent molecules are filled in the voids/holes. During the aging step, the continuation of the condensation reaction leads to the strengthening of the structure. Further washing and drying steps, the gel can be transformed into the final solid-state product *via* eliminating the encapsulated species. Following the conventional preparation method, initial hydrolysis and condensation of the inorganic precursors can happen successively or synergistically. The property of the component formation can be established by the element, ligand constituting the inorganic precursor, and the reaction conditions [100–103]. Notably, the resultant aerogels are substantially different from the similar structures produced by the conventional methods. In the scCO_2_-assisted drying process, the pressurized scCO_2_ permeates through the gel pores or around the surface of materials and eliminates the added organic solvent. A great deal of scCO_2_ flush often leads to the thorough removal of the added organic solvent in the various types of processed materials, which are of particular interest in diverse fields, specifically medicine. Further, the existed scCO_2_ in the interior or surface of the materials can be rapidly eliminated by facile decompression while processing, with minimal influence (shrinkage, collapse, or accumulation) on the porous architectures due to the near-zero surface tension of scCO_2_. Notably, the recovered CO_2_ can be recycled for the consequent synthesis, leading to no wastage of this eco-friendly solvent.

**Figure 7.**
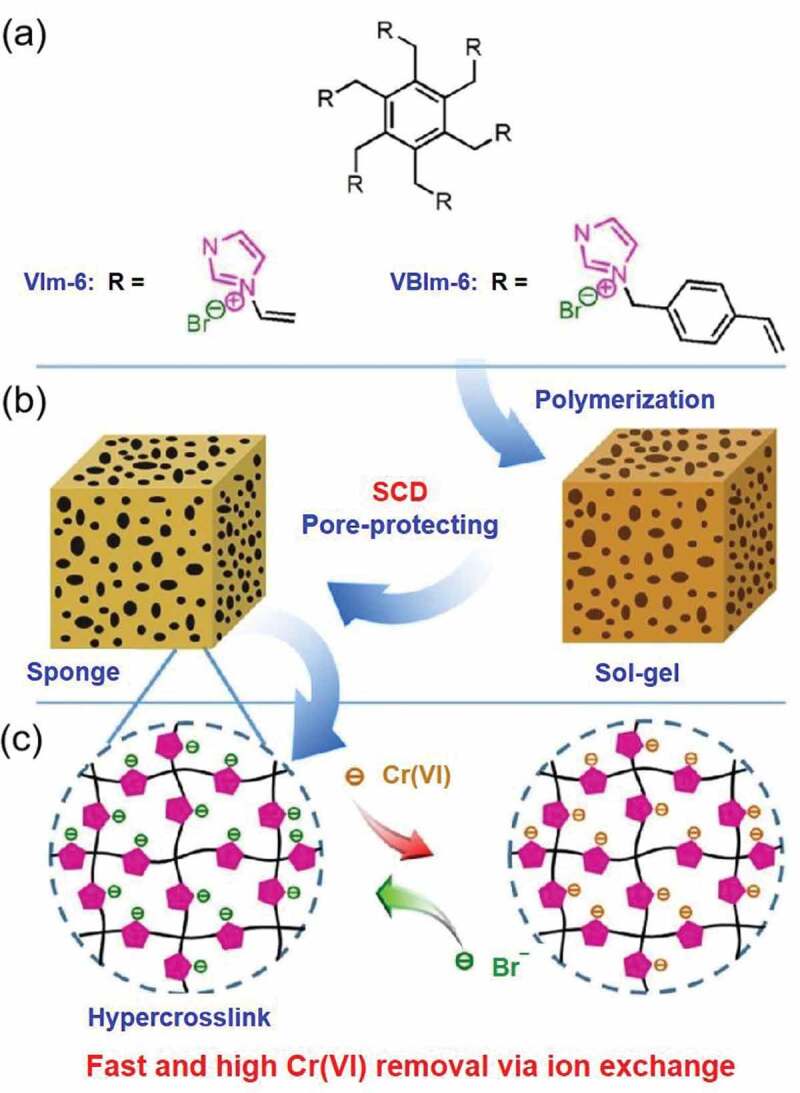
Schematic illustration of (a) monomers VIm-6 and VBIm-6, (b) hypercrosslinked MPILs prepared by the scCO_2_ drying process, and (c) Cr(VI) removal and reabsorption procedure in water over the MPILs. Reproduced with permission from Ref [105]. Copyright 2019, Elsevier

Considering these aforementioned advantages, the scCO_2_ drying process and its advancements have been employed to prepare different types of aerogels, including inorganic-based aerogels. In a case, Zhang and colleagues prepared carbon-coated TiO_2_ (TiO_2_@C) aerogels, in which the oxidizing precursor was subjected to a scCO_2_ drying process and direct carbonization under inert gases [104]. In general, the fabricated scCO_2_-assisted aerogels based on TiO_2_@C presented a large specific surface area with small anatase nanocrystals, which were conveniently enclosed in the filmy carbon layers. In addition, this synthetic method could be deemed as a unique design and simple and convenient procedure for the production of well-distributed carbon-coated metal oxide aerogel anode for lithium-ion batteries. Indeed, the scCO_2_ process-assisted fabrication of aerogel materials can not only provide a larger specific surface area but also bring more adsorption and active sites compared to those of the bulk precursors. In an instance, Xie and colleagues fabricated a series of poly(ionic liquid)s (PILs) based on hypercrosslinked imidazolium with varied ion contents, crosslinker lengths, and textural properties (Figure 7). Among them, PVIm-6 prepared by scCO_2_ drying process displayed excellent textural possessions, such as rich porous structure, large pore volume, and enormous specific surface area, facilitating the exposure of high density of exchangeable Br^−^ in PVIm-6. These consequences had resulted in improving the mass transfer during the ion exchange for Cr(VI). Surprisingly, PVIm-6 showed virtuous selectivity for Cr(VI) among various competing anions, in which the experimental data indicated an 84.5% of utilization coefficient of the adsorption positions and a 236.8 mg·g^−1^ of Cr(VI) adsorption volume. Moreover, the authors demonstrated that these scCO_2_-assisted composites were highly effective for Cr(VI) removal under a broad pH range of 2 to 12, which could be of good practical applicability in exploring their potential towards industrial application prospects [105].

**Figure 8.**
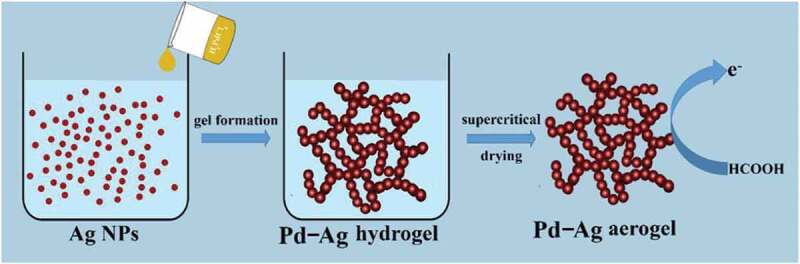
Schematic illustrating the production and application of Pd-Ag aerogel. Reproduced with permission from Ref [106]. Copyright 2019, Elsevier

In another instance, Douk and coworkers prepared the Pd-Ag and Pd-Ir aerogels under the controlled critical conditions of the scCO_2_ drying-assisted galvanic displacement of sacrificial silver and iridium nanoparticles by employing H_2_PdCl_4_ (Figure 8) [106,107]. The designed aerogels reflected several advantages in the oxidation of formic acid and ethanol. After the scCO_2_-assisted drying process, the resultant porous character resulted in a larger specific area over the precursor, leading to the rapid mass transfer rate through the ample portiforium. In addition, the exceptional feature of macroporous architectures in this kind of aerogel assured easy access of reaction substances to the active sites. Owing to the distinctive framework of synthetic aerogel, these designed aerogels based on scCO_2_-assisted drying offer other advantages like meso-and macroporous properties. In a case, Chen and coworkers produced Co-Pt/MgO-Al_2_O_3_ polymetallic aerogel *via* a sol-gel-assisted scCO_2_ drying method [108]. In another case, Matsuyama and colleagues generated the AgNPs-encapsulated cellulose nanofibers (CNFs), in which the ethanolic solution of CNF hydrogel was mixed with AgNPs dispersions and then dried by using the scCO_2_ process [73]. Notably, these designed fibers showed better distribution and remarkable antimicrobial activity over the similar inorganic constructs obtained by the conventional drying methods. It should be noted that this process can promote the active metal diffusion ability and induce good interaction between the other active metals. The scCO_2_ drying technology generally employs core equipment of autoclave with general working pressure as high as 7 ~ 20 MPa. Notably, this special equipment system is highly complicated, and the operational, as well as maintenance costs are even higher compared to the normal pressure drying equipment. Comparatively, the production competence, and safety of scCO_2_-based drying technology are reliant on this core equipment.

### 2.5 Synthesis and processing of MOF composites

Although diverse types of materials of interest based on organic- and inorganic-based constructs have been explored, the search for new materials and their prototypes with critical advancements have been continuing in their corresponding fields of interest. Among such improvements, the fabrication of MOFs, inorganic-organic composite, has built enormous hope and scope in researchers in various fields [109,110]. MOFs, one among the innovative inorganic-based materials, have garnered enormous attention from researchers in recent times, owing to their diverse compositions yielding composite type arrangements and addressing the limitations of both organic-, and inorganic-based materials, among others. Despite the exceptional features and advantageous characteristics of scCO_2_ in diverse inorganic-based nanoarchitectures, some specific properties of CO_2_ make it unsuitable for the processing of MOFs, such as no dipole moment and low specific inductive capacity as well as polarizability. As a result, the building-up of MOFs by employing the scCO_2_ is highly challenging due to the poor solubility of the metal reagents and organic ligands. Typically, scCO_2_ has been utilized for two important criteria. One of them is the preparation of MOFs with new performance, while the other is promoting the performance of MOFs, also known as the activation process. The whole process of MOF synthesis in scCO_2_ includes various steps of the phase transformation of CO_2_, complete removal of the organic solvent remaining in the porous structures, and the transformation process. The sequential steps result in improved properties like zero surface tension and excellent capillary forces, which can substantially avoid the collapse or tunnel blockage of MOFs [111]. Several other methods applied in the preparation of meso- and micro-porous MOFs include the surfactant-template method [112,113] and the ligand extension method [114,115], Comparatively, the application of the expanded CO_2_ for MOF formation is more advantageous as it is template-free and more controllable, involving no additional more-extended ligands. It should be noted that the application of the expanded CO_2_ can significantly quicken the MOF formation, ensuing in the quick recovery of the resultant product through CO_2_ extraction.

Currently, using the scCO_2_ as the activation medium has already become a novel and effective step for the fabrication of MOFs with improved physicochemical properties and performance attributes. Moreover, the activation process is an effective way to enlarge the interior surface area of MOFs, which is of particular interest for varied applications. In a case, Hupp and colleagues prepared the innovative scCO_2_-activated Zn-MOFs with a higher BET specific area compared to the MOFs activated by traditional activation methods, like thermal treatment and solvent exchange [116]. In another case, He and colleagues utilized an effective route for the scCO_2_ activation of γ-cyclodextrin MOFs (CD-MOFs), keeping the organic solvent residual off tunnel block and also avoiding the collapse of the designed MOF frameworks [27]. Moreover, these MOFs resulted in improved textural properties of high pore volume and large specific surface area. The scCO_2_ activated CD-MOFs were applied to explore them as drug carriers for some of the water-insoluble drugs. For instance, honokiol (HNK) was impregnated into the MOFs pores, achieving higher dispersibility of drug, and higher cargo loading, comparing to those of the conventional methods. The authors demonstrated that this method could significantly maintain structural integrity and offer improved performance of MOFs. In addition to drug delivery, this eco-friendly technology has dramatically expanded the application of MOFs in various fields, such as catalysis. In another instance, Cassandra and coworkers prepared the embedded polyoxometalates (POMs, H_5_PV_2_Mo_10_O_40_) in the mesoporous NU-1000. The resultant MOFs were activated by aerobic oxidation of 2-chloroethyl ethyl sulfide (CEES, mustard gas simulant) in cyclohexane using O_2_ and isobutyraldehyde as the oxidant and sacrificial reductant, respectively [100]. Immobilization in the NU-1000 MOFs would improve the diffusion of the substrate since pores would not be obstructed with POM and favor interactions between the MOF and POM. Therefore, the POMs maintained their catalytic efficacy and permitted for recycling of the material. Further, the MOFs presented an enhanced performance by adopting the scCO_2_ activation [26], in which the resultant properties have been prolonged to assist their applicability in drug delivery [101–103], gas storage [117], and gas/ liquid adsorption [118].

In addition to activation, the MOFs can be fabricated in CO_2_/scCO_2_ systems. In a case, Elizabeth and colleagues illustrated an inventive method for the rapid preparation of MOFs in a scalable continuous-update scCO_2_ container [119]. This technique resulted in the Zr-based UiO-66 MOFs at a preparation speed of 104 g·h^–1^ under 3 seconds. Using this method, the yield of MOF in scCO_2_ was greatly improved, and the application of this method has been expanded. In another case, Liu and colleagues prepared the Mn_3_(BTC)_2_-MOF *via* introducing CO_2_ and water emulsion into the synthesis system [120]. The fabricated emulsion by blending the mixture of water, CO_2_, and MOF at 300 K was highly stable against accumulation. The MOF nanostructures assembled at the CO_2_-water boundary built a durable, protective parclose around the dispersed droplet, thus inhibiting the droplet coalescence effectively. Zhang and colleagues proposed the CO_2_-induced MOF synthesis with highly porous network-like structures by utilizing CO_2_ as a tunable solvent. In an attempt to fabricate scCO_2_-assisted micro/mesoporous MOFs, the mesocellular Cu_3_(BTC)_2_ was initially synthesized in CO_2_-expanded N, N-dimethylformamide (DMF) system at 30°C, which was further applied for the synthesis of the mesoporous structure of Cu-MOFs, resulting in the high porosity (13–23 nm).

**Figure 9.**
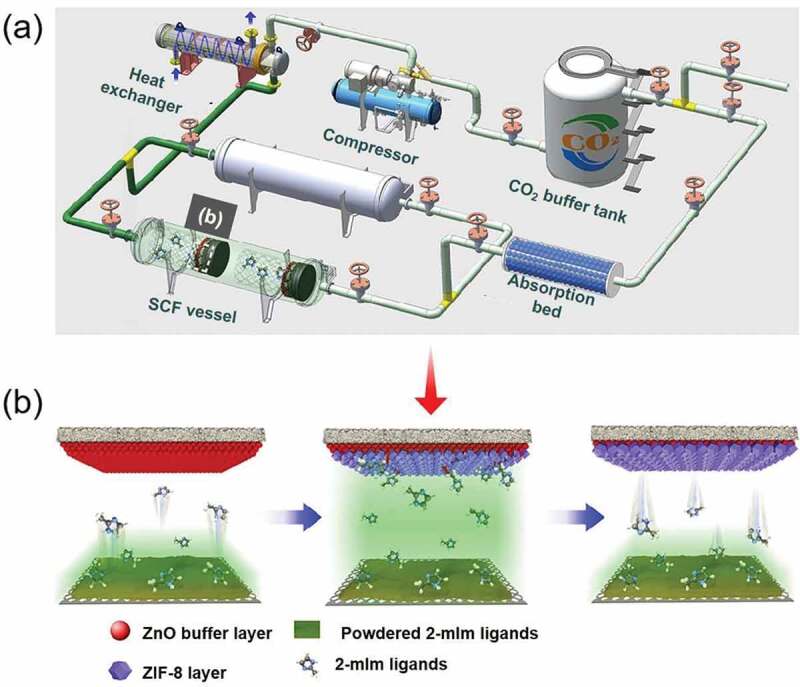
(a) Schematic illustrating the proposed scCO_2_ processing of ZIF-8 membrane, (b) Schematic illustrating the in situ growth of ZIF-8 membrane in scCO_2_. Reproduced with permission from Ref [122]. Copyright 2020, American Chemical Society

**Figure 10.**
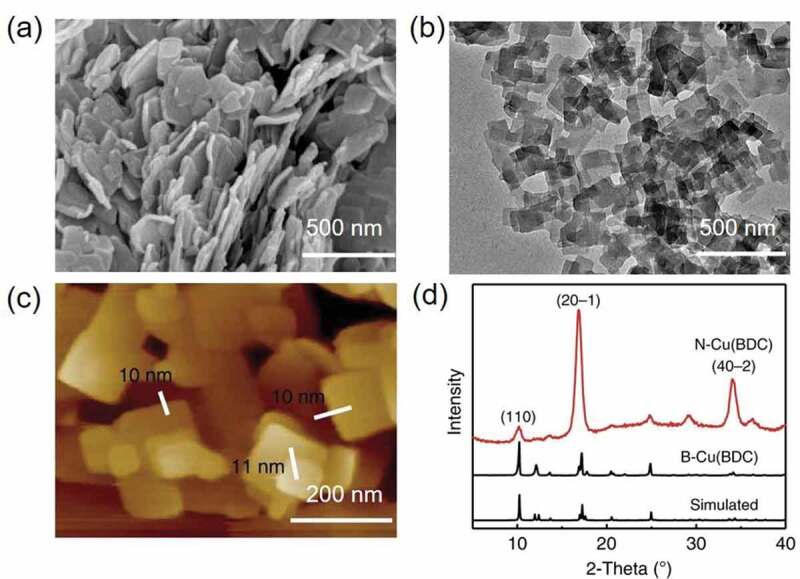
(a-c) SEM, TEM, and AFM images of N-Cu (BDC) synthesized in scCO_2_; (d) XRD patterns of Cu (BDC) nanosheets, bulk Cu (BDC), and simulated XRD pattern of bulk Cu(BDC). Reproduced with permission from Ref [123]. Copyright 2020, Nature publishing group

Notably, the highly advantageous textural properties of MOFs can be conveniently tuned by monitoring the CO_2_ pressure [121]. In addition, some low-dimensional MOF materials can be prepared using the eco-friendly scCO_2_ system. In a case, Liu and coworkers prepared a MOF-based membrane-like structure using a typical scCO_2_ device set-up. The complete preparation method was illustrated in several steps. An even ZnO precursor layer was initially precipitated on the substrate, which not only markedly improved the formation of ZIF-8 structure but also deemed as an only metal source of ZIF-8 membrane. Further, the ZnO precursor layer-modified substrate was positioned along with a given amount of 2-methylimidazole (2-mIm) in a customized semi-permeable cylindrical chamber. Then, sprayed CO_2_ into the chamber was spontaneously transformed to scCO_2,_ resulting in supercritical growth. Finally, the recovery of both discharged CO_2_ and unreacted 2-mIm ligands was conveniently performed (Figure 9) [122]. Notably, the intercrystalline defects could be productively reinforced through this process, leading to the formation of complete ZIF-8 membranes with paraffin/olefin (C_3_H_8_/C_3_H_6_) departure efficiency. To enhance the unsaturated metal active sites on the CuBDC surface, Zhang and colleagues proposed a fabrication strategy using CO_2_ as a coating agent towards directly generating CuBDC nanosheets. (Figure 10). The nanosheets were of an ultrathin thickness (∼10 nm), asmall lateral size (∼100 nm), and a great deal of unsaturated coordination metal sites on the surface [123]. Owing to these distinctive characteristics, the MOF nanosheets with many active sites are conveniently utilized for the oxidation of alcohols with significantly improved catalytic performance. On the other hand, the scCO_2_ synthesis path can significantly improve the preparation efficiency of MOFs. In a case, López-Periago and colleagues produced the 1D Cu (II) MOFs using the scCO_2_ route at 60°C and 20 MPa with productivity of nearly 100% [124].

Although the scCO_2_ has been utilized for the fabrication of MOFs towards improved performance in catalysis and environmental-related applications, these scCO_2_-assisted MOFs have not yet applied in the field of life sciences, particularly in biocatalysis, biosensing, and regenerative medicine, which may become very distinctive and effective directions in the future. Moreover, we believe that the highlighted scCO_2_-fabrication methods utilized in the current research of structures will inspire the researchers in search of new directions and endeavors for peoples’ health and sustainable development.

## 3. Summary and outlook

In summary, this review has highlighted and discussed various fabrication approaches based on the scCO_2_-assisted technology to manufacture, stimulate, and characterize inorganic nanomaterials for catalysis, and biomedicine, energy, as well as environment fields. In general, these fabricated scCO_2_-assisted nanomaterials exhibited enhanced physicochemical properties (e.g. specific area, particle size, amorphous, and distribution) compared to their bulk counterparts, typically leading to improved performance in their respective fields. In most of the instances, SAS-based methods were employed for the preparation of the metal oxide as the reaction precursors and reactants. Moreover, this article outlined a brief perspective of their application in photochemical catalysis and nanozymes catalysis, including the simulating enzyme activity and light absorptivity of the metal oxide nanoparticles. Although the unique advantages of scCO_2_-based processes have been explored widely, it is much less used in the fabrication of inorganic nanomaterials over other conventional methods, such as chemical precipitation, microemulsion, and hydrothermal processes.

Notably, the scCO_2_-assisted technology in the polymerization of materials offers a great potential to be a moderate and green alternative for synthesizing inorganic nanomaterials. By applying this approach, the use of organic solvents would be drastically reduced or completely avoided. However, most of the preparation methods of inorganic-based nanomaterial presented in this article need further research, especially in establishing fundamentals in studying and applying them in diverse fields. First, several attributes are required to be explored towards the multi-scale model development, such as understanding the relationship between solute-solvent interactivity, synthesis procedure in complex reaction systems, and the relationship between composites growth kinetics and fluid dynamics in scCO_2_. Second, existing research based on the laboratory scales conventionally should be further extended. Although enormous progress in the synthesis of scCO_2_-assisted inorganic materials has been made in the last decade, scale-up and continuous process design are required for industrial applications of scCO_2_ for the preparation of inorganic materials, providing a possibility for large-scale industrial catalyst production.

Owing to the development of diverse nanomaterials based on the scCO_2_ technology with efficient light absorption and photocatalysis ability, several types of inorganic-based materials will undoubtedly find widespread applications in the fields of nanocatalysis and nanomedicine. Amongst all, the SCD method is one of the best processes for the synthesis of supported nanoparticles according to nanoparticle size control and dispersion. Due to the high diffusion property of scCO_2_, the facile nature of the preparation method, the emergence of many lubricants, and the high performance of catalysts, it is expected to be applied as the large-scale preparation method of industrialization. Furthermore, phase engineering of transition metal dichalcogenides, as well as 2D heterostructures manufactured adopting ultrathin 2D nanomaterials as encouraging building blocks, can also be realized conveniently with the assistance of scCO_2_. In addition, numerous catalysts fabricated using the scCO_2_ drying approach show the importance and suitability of obtaining nanostructures with enhanced catalytic performance. This green, energy-saving process is expected to be an alternative to other drying methods. Moreover, it is anticipated that the scCO_2_ has been utilized for improved performance or to prepare MOFs with new performance. We also believe that this eco-friendly SCF technology will become a promising method for preparing inorganic materials in the future.
